# Integration of Raman spectra with transcriptome data in glioblastoma multiforme defines tumour subtypes and predicts patient outcome

**DOI:** 10.1111/jcmm.16902

**Published:** 2021-11-12

**Authors:** Pierre‐Jean Le Reste, Eleftherios Pilalis, Marc Aubry, Mari McMahon, Luis Cano, Amandine Etcheverry, Aristotelis Chatziioannou, Eric Chevet, Alain Fautrel

**Affiliations:** ^1^ Department of Neurosurgery University Hospital Rennes France; ^2^ INSERM U1242 University of Rennes Rennes France; ^3^ REACT – Rennes Brain Cancer Team Rennes France; ^4^ e‐NIOS PC Kallithea‐Athens Greece; ^5^ IGDR CNRS University of Rennes Rennes France; ^6^ Centre de Lutte Contre le Cancer Eugene Marquis Rennes France; ^7^ H2P2 Platform UMS CNRS 3480 – INSERM 018 University of Rennes Rennes France

**Keywords:** data integration, glioblastoma, Raman spectroscopy

## Abstract

Raman spectroscopy is an imaging technique that has been applied to assess molecular compositions of living cells to characterize cell types and states. However, owing to the diverse molecular species in cells and challenges of assigning peaks to specific molecules, it has not been clear how to interpret cellular Raman spectra. Here, we provide firm evidence that cellular Raman spectra (RS) and transcriptomic profiles of glioblastoma can be computationally connected and thus interpreted. We find that the dimensions of high‐dimensional RS and transcriptomes can be reduced and connected linearly through a shared low‐dimensional subspace. Accordingly, we were able to predict global gene expression profiles by applying the calculated transformation matrix to Raman spectra and vice versa. From these analyses, we extract a minimal gene expression signature associated with specific RS profiles and predictive of disease outcome.

## 1 INTRODUCTION

Glioblastoma multiforme (GBM; WHO Grade IV), the most aggressive form of primary brain tumours, harbour a dreadful prognosis despite invasive treatments including maximal safe resection, radiotherapy and chemotherapy.^1^ Although all glioblastomas share common histopathological characteristics, they form a highly heterogeneous group of tumours in terms of underlying molecular and genetic alterations. Some alterations already were linked to strong therapeutic and survival outcomes, such as the methylation of the MGMT promoter and the presence of mutations in the isocitrate deshydrogenase (IDH) gene.^2^, ^3^

As such, one of the keys of future targeted therapies is the optimization of the stratification of patients and tumours. Currently, two main molecular sub‐classifications of glioblastomas are widely used. The first is based on the presence of a pre‐existing low‐grade tumour (primary vs. secondary GBM, correlated with IDH mutations) whereas the second derives from a multiparametric clustering, mostly based on genomic data, which differentiated four subtypes of GBM (mesenchymal, pro‐neural, neural and classical).^3^, ^4^ This classification is strongly correlated with clinical features, but the limited therapeutic arsenal against GBM, as well as the genetic information, it requires preclude it from being used in clinical settings. Furthermore, no metabolic data have been so far used in either classification, thus, limiting a complete exploration of tumour physiology.

Raman or vibrational spectroscopy (RS) is a non‐invasive, label‐free technique allowing chemical analysis based on the analysis of the reflection of a monochromatic light on a sample. As the wavelength shift of the scattered light is correlated with some molecular structural properties, RS is able to provide an immediate chemical fingerprint of a sample. RS is widely used in chemistry, but its application in biology has long been restricted due to technical limitations and issues in processing complex spectra related to the multiplicity of different chemical compounds found in biological samples such as tumours.^5^, ^6^ Two main spectroscopy techniques were described in the literature (Raman and infrared spectroscopy), with highly variable conditions across studies, and numerous subtypes of technologies.^7^ Raman and IR spectroscopy might provide complementary information, considering that they do not assess the same physical phenomena. However, one of the main advantages of RS is the absence of need for sample preparation, thus, allowing direct analysis of tissues. Several studies showed that RS is able to discriminate normal brain from tumour tissue with high specificity, and even differentiate different types of primary brain tumours.^8^, ^9^, ^10^ Recent developments of miniaturization now enable RS to be performed during surgical removal, thus, providing continuous information about the nature of the tumour and resection margins.^8^, ^9^, ^11^ However, if the diagnostic power of RS has been studied considering the whole spectra, no detailed analysis of the data provided by individual peaks has been performed to our knowledge.

Herein, we hypothesized that tumour RS might correlate with genomic data and, therefore, allow the rapid and accurate determination of tumour features and prognosis. To test this hypothesis, we utilized transcriptome data on RS‐based GBM clustering. We identified a RS‐based signature that correlates with the expression of specific genes and predicts specific tumour features associated with aggressiveness. We discuss how this tool could be used in a clinical context.

## 2 MATERIALS AND METHODS

### 2.1 Samples processing and transcriptomic data

Tumour samples were frozen after surgical resection performed at the department of Neurosurgery of Rennes. Informed consent was obtained in accordance with the French legislation and biological samples were stocked in the local biobank (Centre de Ressources Biologiques Santé of Rennes, BB‐0033‐00056). The research protocol was conducted under French legal guidelines and fulfilled the requirements of the local institutional ethics committee. Normal brain samples came from cortectomies. Histopathological analysis was carried out by the local histopathology department. Different cohorts of patients were used: a cohort comprising different types of gliomas for the exploratory phase, a previously published cohort (GBM‐MARK) comprising transcriptomic analysis and clinical annotation^12^ for the main analysis, and a last set of samples were used for a validation phase of the Raman analysis. Clinical and histological data are showed in Table 1. Complementary survival data were extracted from the TCGA cohort available through the TCGA Data Portal (https://portal.gdc.cancer.gov).

**TABLE 1:** Clinical characteristics of glioblastoma patients

Characteristics	RAMAN cohort
Age, years	
Median	58
Range	36–75
Age, *n* (%)	
≤50	7 (18%)
>50	31 (82%)
Gender, *n* (%)	
Women	10 (26%)
Men	28 (74%)
KPS	
Median	90
Range	60–100
Location of the tumour, *n* (%)	
Frontal	19 (50%)
Temporal	14 (37%)
Parietal	3 (8%)
Occipital	2 (5%)
Type of surgery, *n* (%)	
Partial resection	16 (42%)
Complete resection	22 (58%)
Nb of TMZ cycles	
Median	6
Range	1–12
PFS (months)	
Median	10.7
Mean	13.2
95% CI	11.4–14.6
OS (months)	
Median	17.1
Mean	19,9
95% CI	17.4–20.6

Abbreviation: KPS, Karnofsky performance status at diagnosis.

### 2.2 Raman spectroscopy

Raman spectra were acquired with a ThermoFisher Raman Microscope DRX2. Frozen samples were cut in slices of 30 µm thickness. Three fields of acquisition were randomly selected in non‐necrotic areas (100 µm × 100 µm, 121 spectra by field). The acquisition conditions were as follows: 532 nm laser, laser power 3.5 mW, exposure time 0.2 s, 45 iterations, pinhole diameter 25 µm. Each sample's final spectrum corresponded to a mean of all acquired spectra.

### 2.3 Statistical analyses

#### 2.3.1 Processing of Raman spectra

Raman spectroscopy data were analysed with R ChemoSpec 5.0.229 package.^1^ Raw signals for 30 samples were imported and processed for (i) Quality control, detection and removal of outliers, (ii) baseline correction (removal of background effects) (Andreas F. Ruckstuhl, Matthew P. Jacobson, Robert W. Field, James A. Dodd's algorithm based on LOWESS and weighted regression), (iii) hierarchical cluster‐based peak alignment,^2^ (iv) binning (smoothing of signals by summing every *n* = 20 intensity values), (v) normalization using the Probabilistic Quotient Normalization (PQN) method.^3^

#### 2.3.2 Clustering of Raman spectra

Model‐based clustering of spectra was performed, using the R package mclust in order to find the optimal number of clusters. The selected optimal model comprised 2 clusters maximizing the distance amongst distinct groups. Each spectrum was then labelled according to its belonging to either group (Raman Group 1/Group 2).

#### 2.3.3 Differential gene expression analysis

(i) Processing of Raman spectra: Raman spectroscopy data were analysed with R ChemoSpec 5.0.229 package.^1^ Raw signals for 30 samples were imported and processed for (a) Quality control, detection and removal of outliers, (b) baseline correction (removal of background effects) (Andreas F. Ruckstuhl, Matthew P. Jacobson, Robert W. Field, James A. Dodd's algorithm based on LOWESS and weighted regression), (c) hierarchical cluster‐based peak alignment,^2^ (d) binning (smoothing of signals by summing every *n* = 20 intensity values), (e) normalization using the Probabilistic Quotient Normalization (PQN) method.^3^ (ii) Clustering of Raman spectra: Model‐based clustering of spectra was performed, using the R package mclust to find the optimal number of clusters. The selected optimal model comprised 2 clusters maximizing the distance amongst distinct groups. Each spectrum was then labelled according to its belonging to either group (Raman Group 1/Group 2).

#### 2.3.4 Differential gene expression analysis

Differential gene expression analysis was performed on microarray gene expression data (quantile normalization) using the Rank Products method (R package RankProd), which performs multiple permutations, in order to infer robustly the statistical significance of the expression change by measuring the variation of the rank of each gene. Gene consistently highly ranked in multiple comparisons between the two Raman groups were extracted as significantly differentially expressed (671 genes).

#### 2.3.5 Multiple factor analysis combining gene expression with Raman spectroscopy

Multiple Factor Analysis (MFA) is a variation of Principal Component Analysis (PCA) suitable for heterogeneous variables by projecting them into a common high‐dimensional space.^13^, ^14^ In this case, the gene expression values of the 671 differentially expressed genes were correlated with frequencies of Raman spectroscopy. MFA was performed with the R package *FactoMiner*.^15^ The analysis considered only peaks from 600 to 1800 cm^−1^ and 2800 to 3100, which correspond to biologically meaningful signal ranges. MFA produced composite Eigen vectors (linear combinations of variables) consisting of Raman peak frequencies and gene symbols. Table 2 shows the prioritized genes using as ranking measure their contribution to the first two Eigen vectors (principal components). The top 36 genes were selected as the minimum subset that maximizes the distance between the two clusters.

**TABLE 2:**
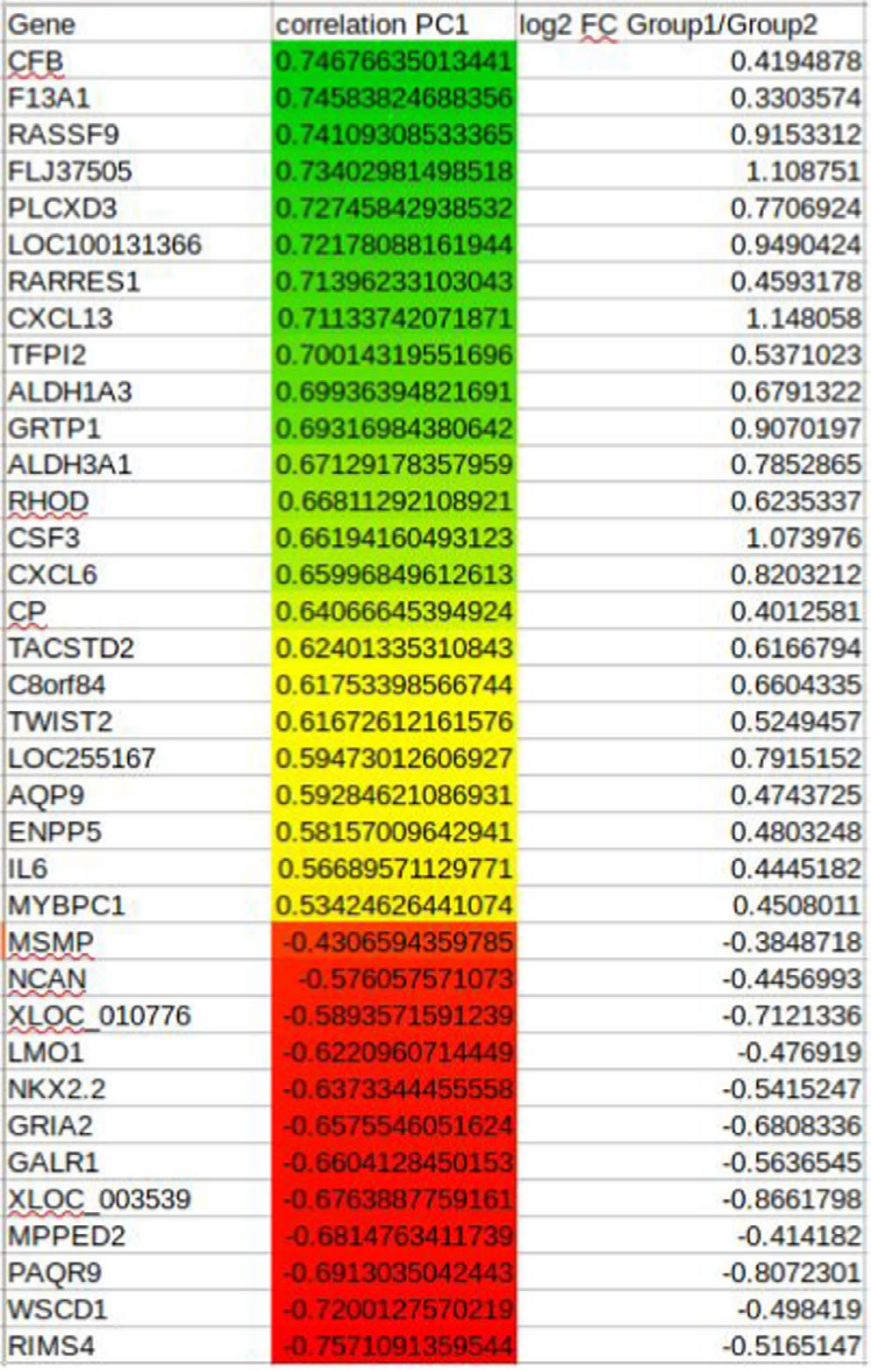
List of prioritized genes according to the first two Eigen vectors (principal components) with Raman group 1 (green), genes are strongly correlated with the group I (yellow) and group 2 (red)

#### 2.3.6 Survival analyses

We performed a cross‐study analysis to assess the prognostic value of the RS‐based signature. Gene expression was used as a predictor and survival time (in months) as the response. We used the TCGA cohort (Affymetrix microarray data) to train the prognostic model. We performed univariate Cox regression analysis on the expression of the genes correlated with the RS‐based classification of GBM and selected those with a *p*‐value threshold at 0.05. We then performed multivariate Cox regression analyses on these genes to create a gene‐based survival model. Patients were then ranked according to their risk score. The optimal risk cut‐off was assessed and used for the stratification of patients into two groups: low risk of death and high risk of death. The Kaplan–Meier method was used to estimate the survival distributions. Log‐rank tests were used to test the difference between survival groups. Analyses were carried out with the *survival* R package. The validation of the prognostic model (same coefficients and cut‐off) was performed on the GBM‐MARK cohort, a technically (Agilent microarray data) and biologically independent cohort.

### 2.4 Histochemistry and immunohistochemistry

Paraffin blocks corresponding to the tumour samples analysed using Raman were processed for immunohistochemistry (IHC). IHC were carried out on the H2P2 core facility. The sections were incubated 1 h at room temperature with anti‐IBA1 (1:10,000 dilution; proteintech). Immunostaining was carried out using the discovery ultra (Ventana Medical Systems) with the Rhodamine kit (a “biotin‐free” system using multimer technology, Roche) and a Tris borate EDTA pH8 buffer for antigen retrieval. Sections were converted on to digital slides with the scanner Nanozoomer 2.0‐RS and immunostaining were quantified with the NIS software (Nikon).

## 3 RESULTS

### 3.1 Histological classification of primary brain tumours using Raman spectroscopy

**FIGURE 1:**
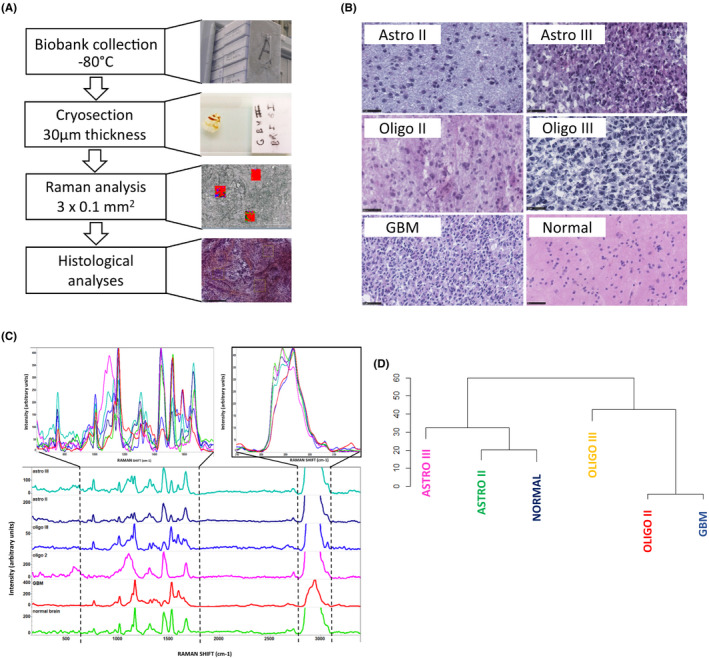
Raman spectroscopy‐based classification of brain tumours. (A) Schematic representation of the analytical pipeline. (B) H&E stained and Histological analysis of 5 tumour types including grade II and III astrocytomas, grade II and III oligodendrogliomas and glioblastoma multiforme (GBM). Bar = 50 μm. (C) Raw Raman spectra of various primary brain tumours (top) and deconvoluted spectra per tumour type: grade III astrocytoma (cyan), grade II astrocytoma (dark blue), grade III oligodendroglioma (blue), grade II oligodendroglioma (pink), GBM (red) and normal brain (green). (D) Hierarchical clustering from Raman spectra of several tumours of each group

Our initial objective was to evaluate whether Raman spectroscopy (RS) was a relevant tool to analyse primary brain tumour in order to classify them and to potentially predict patient outcome. To this end, we designed an experimental approach relying on a collection of frozen tumour samples conserved in our institutional biobank, the “Centre de Resources Biologiques (CRB) de Rennes” and analysed using RS and histochemistry (Figure 1A). Sixteen primary brain tumours (comprising astrocytomas [grade II and III – *n* = 5]; oligodendrogliomas [grade II and III – *n* = 6]; glioblastoma multiforme [GBM – *n* = 5]) and normal brain samples (only for RS) were analysed using histochemistry and RS (Figure 1B,C). Histological analyses revealed several different levels of cellularity, according to tumour types, with oligodendroglioma‐III and GBM those presenting the greater cellularity. Both of them showed also, high level of pleiomorphism and some mitotic figures. Necrotic zones observed in GBM samples were excluded from our study. Matched samples were also analysed using RS and produced various spectra with peaks‐containing regions that were aligned (Figure 1C). Hierarchical clustering allowed us to propose a RS‐based classification for brain tumours. Two well established groups were observed. The first group contained Astro‐III, Astro‐II and normal tissue, Astro‐II resembling more normal tissue than Astro‐III. The second comprised the oligodendroglial lineage, with Oligo II and III as part of the same group. However, GBM shared some characteristics with oligo II suggesting similar metabolic pathways involvement in pathogenesis (Figure 1D). As such, one might conclude from these analyses that RS spectra could sort the tumours based on their nature as determined by histopathology.

### 3.2 GBM stratification using Raman spectroscopy

**FIGURE 2:**
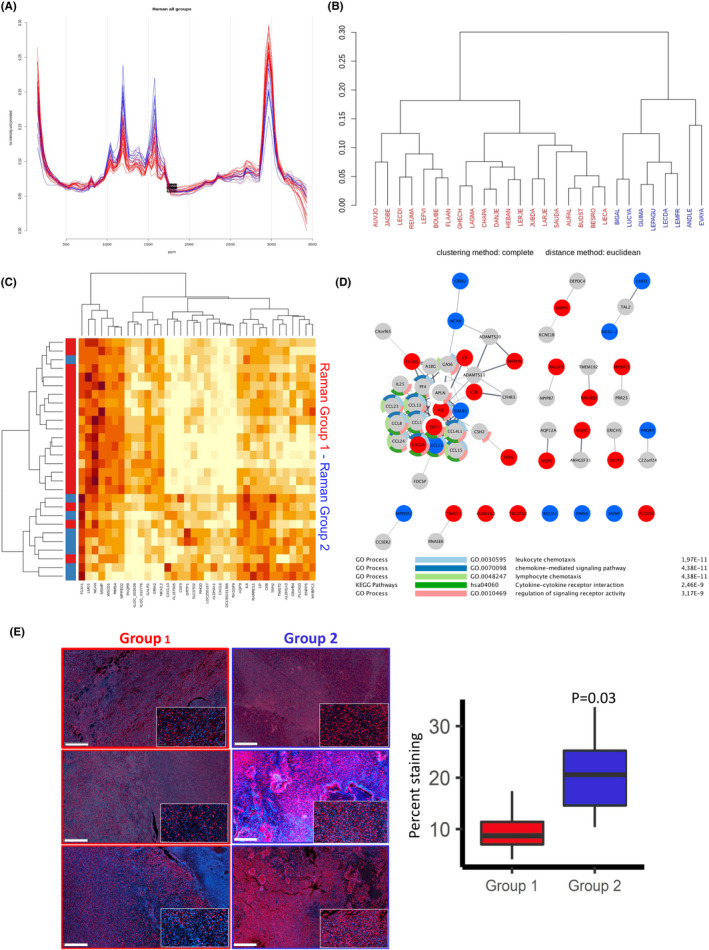
Correlation between GBM Raman profiles and gene expression data. (A) Raman spectra (after baseline correction, smoothing and normalization) corresponding to 28 GBM that segregate in two groups based on their respective profiles (blue and red). (B) Hierarchical clustering of patient tumours based on the corresponding Raman spectra (blue and red). (C) Heat map representation of gene expression profiles matching the groups formed based on Raman spectra. Gene profiles corresponding to the blue and red groups are indicated. (D) String‐derived network comprising genes correlating with Raman Group 1 (red) and 2 (blue). Functional enrichment might be indicative of an immune infiltration in tumours from group 1. (E) Immunofluorescence analysis of tumour sections from group 1 and 2 using anti‐IBA1 antibodies (staining macrophages and microglial cells; left panels, scale bar: 1 mm) and quantitation of the staining (right panel)

To further push this idea, we next sought to test whether the power of RS would be sufficient to identify different groups in GBM, a tumour type known for its high heterogeneity.^16^ To this end, 34 tumours from the GBMmark cohort and 23 other GBM were analysed using RS and this led to the identification of two major RS spectrum profiles (Figure 2A). Lipid, nucleic acid and protein content allows the detection of characteristic RS peaks that corresponded to the vibration/ rotation of functional groups of atoms in the region between 600 and 1800 cm^−1^ (fingerprint) whereas characteristic band of spectral peaks observed between 2800 and 3100 cm^−1^ are attributed to the vibrations of methyl(‐CH3) and methylene (‐CH2‐) groups. When these spectra were used to hierarchically cluster the tumours, two highly distant groups were obtained (Figure 2B). Next, in an attempt to functionally annotate the two groups of tumours, we performed MFA in order to identify the genes whose expression correlated with Raman group 1 and those whose expression correlated with Raman group 2. A total of 36 genes were selected, the expressions of which maximized the distance between the two groups in the clustering analysis (Figure 2C). Of those genes, 24 correlated with group 1 and 12 with group 2 (Figure 2C, Table 2). Their correlation values with the first principal component (PC1) (Figure 2C) highlight the direction in which the expression of the genes shapes the Raman spectrum; a positive correlation value signifies that the upregulation of a gene results in more intense peaks around ~3000 cm^−1,^ and less intense peaks around 1000–1600 cm^−1^, whilst a negative correlation value signifies the exact opposite. Interestingly, tumour clustering based on the expression of these 30 genes almost perfectly recapitulated the segregation obtained using RS data. At last, we functionally analysed the genes identified through this approach and found that genes whose expression correlated with Raman profiles might relate to an altered immune cell infiltration (Figure 2D). This prediction was evaluated using immunohistochemistry with anti‐IBA1 antibodies (for the staining of macrophages and microglial cells) in matched tumour sections and revealed that RS‐based Group2 tumours were more infiltrated by myeloid cells than RS‐based Group1 tumours (Figure 2E), which could be indicative of a differential tumour aggressiveness provided that the infiltrate could be immune promoting or suppressive.

### 3.3 Establishment of GBM RS‐based molecular signatures

**FIGURE 3:**
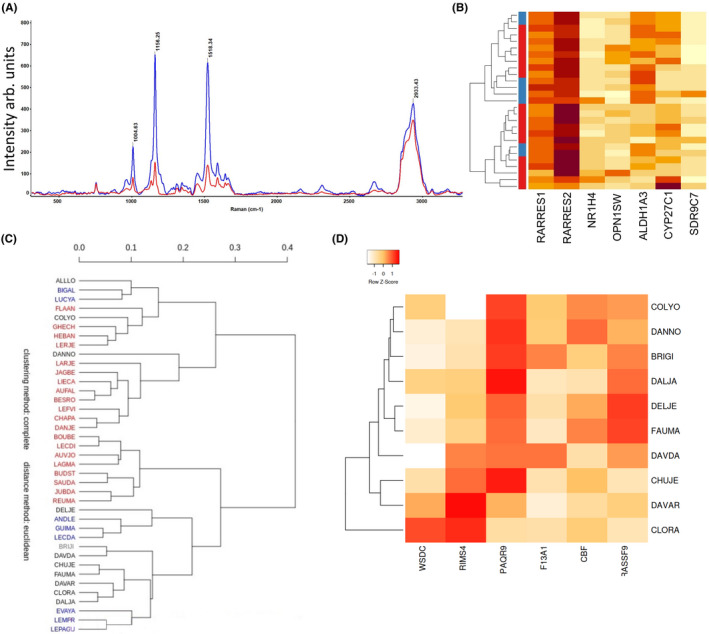
Integration of molecular and Raman signatures highlighting Vitamin A pathway. (A) Average Raman spectra from which average background was subtracted for GBM group1 is indicated in Red and for GBM group2, indicated in Blue. (B) Heat map expression of seven genes, involved in the biosynthesis of vitamin A. (C) Hierarchical clustering of patients GBM mark group1 (red) and group2 (blue) and validation cohort (black). (D) Heatmap expression of six genes, robustly reflecting the classification using RS, and also the validation cohort

Since we established that RS‐based classification of GBM provided a relevant tool to characterize the tumour features, we next sought to further document the links between RS analysis and tumour characteristics. The first step in this initiative was to identify the nature of the peaks marking Group1 and Group2. The most remarkable difference between the spectra identified two groups corresponding to the intensity of the two peaks 1156 and 1518, four times more important in group 1. These peaks are part of the peaks representing the carotenoids.^5^ We also noticed a smaller increase of peak 957 and peak 1004, the latter being a mixed Raman peak, with contribution of carotenoids and phenylalanine (Figure 3A). As the main peaks identified in Group1 corresponded to retinoids, we next evaluated how the expression of genes involved in the biosynthesis of vitamin A was altered in both groups. Interestingly, the expression of seven selected genes involved in this pathway did not yield to a clear segregation of the two groups as performed following RS‐based analysis (Figure 3B). This observation might indicate a non‐transcriptionally dependent regulation of retinoid production in GBM and led us to further inquire the existence of a minimal gene signature correlating with the RS‐based groups. An in‐depth analysis of the data presented in Figure 2 led us to identify six genes (WSDC, RIMS4, PAQR9, F13A1, CBF and RASSF9) whose expression variation robustly reflected the classification obtained using RS. The expression of these genes was then evaluated in a validation cohort of 10 tumours classified using RS (Figure 3C) and confirmed the results obtained on the test cohort (Figure 3D). The approach described above allowed us to demonstrate that RS‐based classification of GBM correlates with specific gene expression signatures and, thus, might have a predictive value regarding tumour outcome.

### 3.4 RS‐based molecular signature and prediction of tumour outcome

**FIGURE 4:**
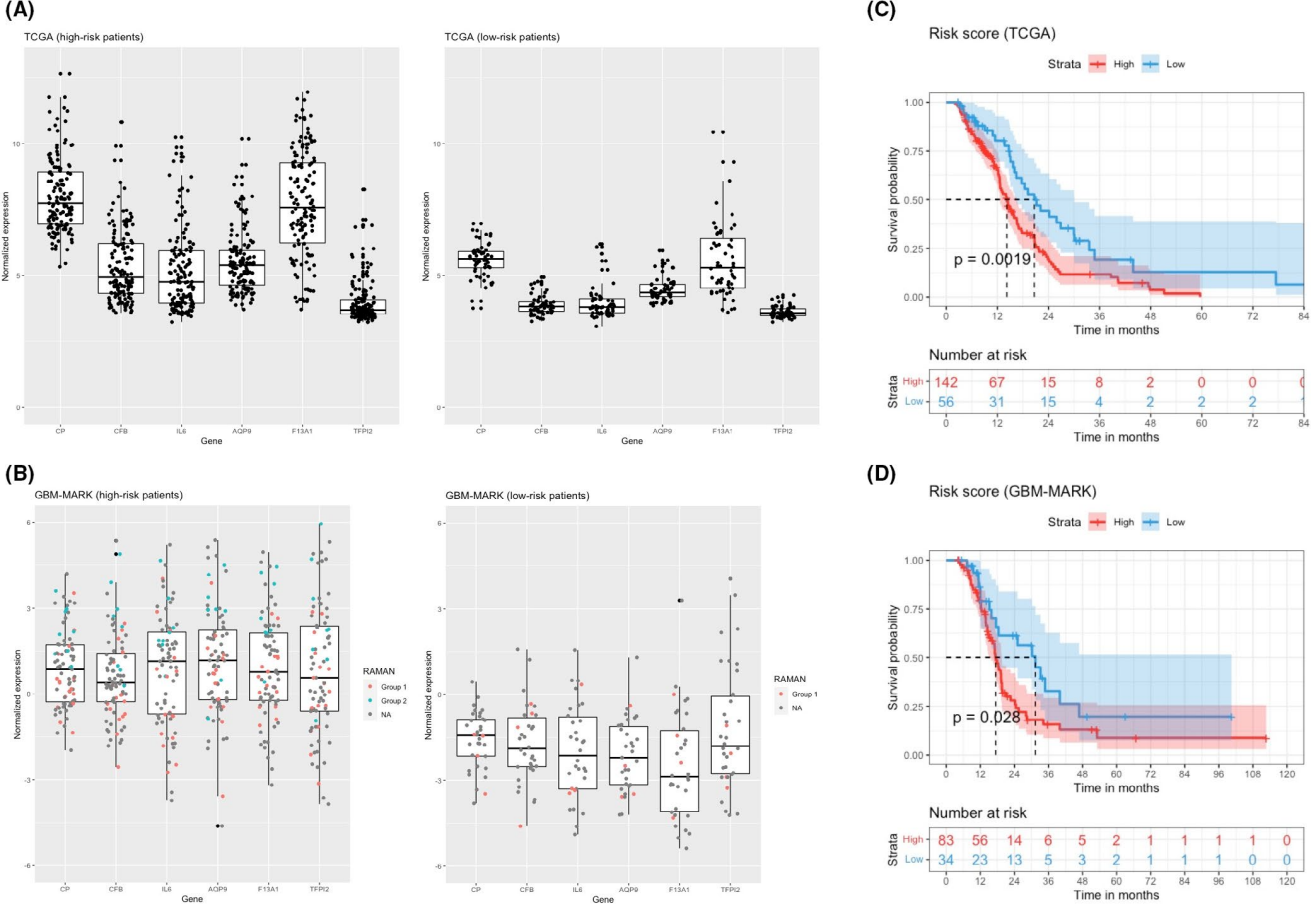
Raman signature and survival prediction. Normalized expression of the genes included in the prognostic model for the TCGA (training) (A) and GBM‐MARK (validation) cohorts (B). In the validation cohort, the RAMAN patient classification is reported in red (group 1) or blue (group 2) dots. Survival analysis in TCGA (C, training) and GBM‐MARK (D; validation) cohorts. The signature‐gene expression levels are reported for the two groups identified by the prognostic model (high risk/low risk). Survival of GBM patients according to the risk score derived from the RS‐based molecular signature. Kaplan–Meier estimates of overall survival in the cohort after subdivision into two groups (low and high risk of death) on the basis of the risk‐score model. The difference in survival between groups is reported (log‐rank test *p*‐value). The shade around the lines represents the 95% confidence interval. Median survival in each group is indicated by the dashed line

To follow up on this analysis, we used the TCGA cohort, the genes included in the prognostic model based on the genes found to correlate with RS showed differential expression between high‐risk and low‐risk patients (Figure 4A). This was also observed in the validation GBM‐MARK cohort (Figure 4B). In the latter cohort, the expression of these signature genes was also significantly lower for patients from the Raman Group 1 compared to those in the Raman Group 2 (Table 3). The multi‐gene survival model based on this molecular signature was used to stratify each cohort into high‐risk and low‐risk patients. In the TCGA cohort, the overall survival (OS) was significantly higher in low‐risk patients (*n* = 56) than in high‐risk patients (*n* = 142): 20.7 months (95% CI, 16.5–30.1) versus 14.2 months (95% CI, 12.6–16.6), respectively; *p* = 0.002 (Figure 4C). This stratification was also observed in the validation cohort with a median OS of 31.4 months (95% CI, 17.5 to not reached) for low‐risk patients (*n* = 34) and of 17.4 months (95% CI, 15.8–19.6) for high‐risk patients (*n* = 83); *p* = 0.03 (Figure 4D). These results indicate that genes from the predictive Raman signature showed higher expression in the Raman group 2 than in the Raman group 1 and their expression correlated with a better prognosis.

**TABLE 3:** Differential expression of the six signature genes between Raman group 1 and Raman group 2 showing the significantly higher expression in Gp2 versus Gp1

Gene	probe	*p*	*p*‐adj
CP	A_33_P3343196	0.0001569146	0.0006460079
CFB	A_23_P156687	0.0018015385	0.0027023077
IL6	A_23_P71037	0.0002153360	0.0006460079
AQP9	A_23_P106362	0.0049949273	0.0064220494
F13A1	A_32_P140139	0.0003993423	0.0008985201
TFPI2	A_23_P393620	0.0066523275	0.0074838684

## 4 DISCUSSION

In a recent study, Riva and colleagues^10^ demonstrated that Raman spectra effectively and accurately discriminated glioma tissue from healthy brain ex‐vivo in fresh samples. This observation, together with the use of intraoperative Raman spectroscopy^9^ to delineate the tumour boundaries, point towards RS as a relevant and versatile tool to classify and characterize brain tumours. In the present study, we have pushed forward this concept by testing the depth of RS‐based brain tumour classification using transcriptome‐related information as our reference. We first showed that RS effectively discriminated different types of primary brain tumours and also at least two major types of the very heterogeneous GBM. As such, we used a well‐defined GBM cohort for which transcriptome data and the corresponding tumour tissues were available to test whether RS profiles corresponded to specific gene expression patterns.

As previously illustrated in many tumour types^5^ and in brain tumours,^6^, ^9^, ^10^ we first evaluated whether RS could discriminate between different types of primary brain tumours and normal brain using frozen sections. Our results indicated that not only RS allowed to discriminate brain tumours from normal tissue but also classify tumours based on their lineage (astrocytoma vs. oligodendrioglioma vs. glioblastoma [GBM]; Figure 1). In our principal components analysis, glioblastoma was classified closer to oligodendroglioma than astrocytomas. Considering the very different oncogenesis mechanisms and prognosis of these two tumoral entities, it is somehow surprising that the Raman spectra showed similar patterns. Two main hypotheses could be considered to explain this phenomenon, first, the existence of a strong oligodendroglial pattern in the GBM samples resulting from a bias in selection, as oligodendroglial patterns can be present in GBM^17^ second, the principal components analysis segregating different spectra due to the similarity of tumour global morphologies, even if the underlying metabolic pathways are quite different. To test the first hypothesis, we performed a histological analysis of our samples, which showed typical GBM morphological characteristics with no strong oligodendroglial patterns. In addition, we restricted our analysis IDH wild‐type samples. However, the participation of an oligodendroglial component cannot be completely ruled out, as some very subtle chemical changes may lead to important spectral changes. Regarding the second hypothesis, the genetic and metabolic drivers are different between GBM and oligodendrogliomas illustrates the complexity of spectral analysis of complex biological samples.

Interestingly, the functional annotation of the gene profiles associated with one of the two groups unveiled an immune cell infiltrated phenotype, which was later validated using immunofluorescence (Figure 2) and also correlated with better survival (Figure 3), which might be indicative of tumour suppressive functions. As such, at present one cannot rule out the fact that the different Raman spectra obtained in group 2 could result from the immune infiltration rather than from tumour cells heterogeneity and remains to be further documented. One of the most striking finding in the analysis of spectra was the occurrence of strong peaks corresponding to carotenoids. We did not identify any variation in the expression of the main components of the canonical carotenoid metabolism pathway; however, two genes related to carotenoid metabolism were found in the 36 genes signature (RARRES1 and ALDH1A3). Indeed, an analysis based on the seven most relevant genes related to carotenoid metabolism failed to match the spectral morphology of the two Raman groups, meaning that post‐translational regulation might occur, as it has been already been showed for the main Vitamin A receptors.^18^, ^19^ Amongst the 36 most significant genes linked to spectral characteristics, we identified two minimal sets of genes with different interests: one set represents the genes that participate the most to the shape of the spectra (WSDC, RIMS4, PAQR9, F13A1, CBF, RASSF9), and one set of highly expressed genes linked to survival (CP, CFB, IL6, F13A1, TFPI2). No major known driver of gliomagenesis was found. Some of the genes related to survival have showed to play a role in the regulation of stem cells and resistance (IL6, CFB), as well as migration and invasion (TFPI2).^20^, ^21^, ^22^ Interestingly, F13A1 was found in both minimal genes set. Factor XIII is an enzyme, which plays a role in the stabilization of fibrin and has been showed to be associated with survival.^23^, ^24^ However, its role in glioblastoma aggressiveness has been scarcely studied. These six genes were expressed at higher level in group 2 than in group 1 and were associated a better survival with group 2 than in group 1 in two independent cohorts of patients (GBM‐MARK and TCGA; Figure 4). As such, one might conclude that RS‐based classification of GBM can document almost in real‐time during surgery not only some characteristics of the tumour (e.g., immune infiltration) but also of its aggressiveness (as indicated by patient survival), both potentially of interest in the decision‐making regarding follow‐up patient handling.

In summary, this study provides the first association between gene expression and Raman profiles associated with tumour phenotypes (e.g., immune infiltrate). The approach presented in this study could, therefore, pave the way for near real‐time intraoperative tumour characterization and could represent a relevant tool for helping in patient management at a very early stage.

## CONFLICT OF INTEREST

EC is a founder of Cell Stress Discoveries Ltd (https://cellstressdiscoveries.com/) and of Thabor Therapeutics. AC is the founder of e‐NIOS Applications PC (https://e‐nios.com/).

## AUTHOR CONTRIBUTION

**Pierre‐Jean Le Reste:** Conceptualization (equal); Funding acquisition (equal); Investigation (equal); Methodology (equal); Project administration (equal); Supervision (equal); Validation (equal); Writing‐review & editing (equal). **Eleftherios Pilalis:** Conceptualization (equal); Investigation (equal); Methodology (equal); Project administration (supporting); Supervision (supporting); Visualization (supporting); Writing‐review & editing (supporting). **Marc Aubry:** Conceptualization (supporting); Investigation (equal); Methodology (equal); Writing‐review & editing (equal). **Mari McMahon:** Investigation (supporting); Methodology (supporting). **Luis Cano:** Investigation (supporting); Methodology (supporting). **Amandine Etcheverry:** Investigation (supporting); Methodology (supporting). **Aristotelis Chatziioannou:** Conceptualization (supporting); Funding acquisition (equal); Investigation (supporting); Methodology (supporting); Project administration (equal); Writing‐review & editing (equal). **Eric Chevet:** Conceptualization (equal); Funding acquisition (lead); Project administration (lead); Supervision (equal); Writing‐original draft (lead); Writing‐review & editing (equal). **Alain Fautrel:** Conceptualization (equal); Investigation (equal); Methodology (equal); Project administration (equal); Visualization (equal); Writing‐review & editing (equal).

## CONSENT FOR PUBLICATION

All the authors read and approved the current version of the manuscript.

## Data Availability

All transcriptomic data are available on open web portals. Raman spectra will be provided upon request.
